# Newborn Length of Stay and Risk of Readmission

**DOI:** 10.1111/ppe.12359

**Published:** 2017-04-18

**Authors:** Katie Harron, Ruth Gilbert, David Cromwell, Sam Oddie, Jan van der Meulen

**Affiliations:** ^1^ Department of Health Services Research and Policy London School of Hygiene and Tropical Medicine London UK; ^2^ UCL Great Ormond Street Institute of Child Health London UK; ^3^ Bradford Neonatology Bradford Royal Infirmary Bradford UK

**Keywords:** Caesarean delivery, length of stay, patient readmission, preterm delivery, hospital records

## Abstract

**Background:**

Evidence on the association between newborn length of hospital stay (LOS) and risk of readmission is conflicting. We compared methods for modelling this relationship, by gestational age, using population‐level hospital data on births in England between 2005–14.

**Methods:**

The association between LOS and unplanned readmission within 30 days of postnatal discharge was explored using four approaches: (i) modelling hospital‐level LOS and readmission rates; (ii) comparing trends over time in LOS and readmission; (iii) modelling individual LOS and adjusted risk of readmission; and (iv) instrumental variable analyses (hospital‐level mean LOS and number of births on the same day).

**Results:**

Of 4 667 827 babies, 5.2% were readmitted within 30 days. Aggregated data showed hospitals with longer mean LOS were not associated with lower readmission rates for vaginal (adjusted risk ratio (aRR) 0.87, 95% confidence interval (CI) 0.66, 1.13), or caesarean (aRR 0.89, 95% CI 0.72, 1.12) births. LOS fell by an average 2.0% per year for vaginal births and 3.4% for caesarean births, while readmission rates increased by 4.4 and 5.1% per year respectively. Approaches (iii) and (iv) indicated that longer LOS was associated with a reduced risk of readmission, but only for late preterm, vaginal births (34–36 completed weeks’ gestation).

**Conclusions:**

Longer newborn LOS may benefit late preterm babies, possibly due to increased medical or psychosocial support for those at greater risk of potentially preventable readmissions after birth. Research based on observational data to evaluate relationships between LOS and readmission should use methods to reduce the impact of unmeasured confounding.

Potentially preventable readmissions, such as for jaundice or feeding problems, make up the majority of early neonatal readmissions.^1^ Theoretically, such admissions could be reduced either through additional support during the newborn hospital stay, or increased levels of follow‐up after discharge (e.g. by midwives or health visitors).^2^ Evidence on safe early discharge is conflicting.^3^, ^4^, ^5^ Much of the evidence comes from the United States, where rates of neonatal readmissions declined following legislation in 1996 mandating insurance for a minimum 48‐h hospital stay for normal deliveries.^6^, ^7^, ^8^ However, several observational studies have demonstrated that decreasing the length of postpartum stay does not increase readmission rates, given adequate postnatal care outside of hospital.^9^, ^10^, ^11^, ^12^ Other studies show associations between shorter newborn length of stay (LOS) and neonatal readmissions and infant mortality.^13^, ^14^, ^15^, ^16^

The lack of consensus can in part be explained by differences in access to care and out‐of‐hospital support available to new mothers in different settings, as well as different definitions of ‘early’ discharge.^5^, ^17^ However, methodological challenges also play a role. Conflicting results from previous studies may be due to the complexities of controlling for risk factors associated with both exposure and outcome.^3^ Evaluating the association between newborn LOS and readmission is complex due to (unmeasured) confounding: babies who stay in hospital for a longer period of time after birth often have serious health conditions that result in higher readmission rates; for babies who are discharged early, higher parental competence may be associated with a reduced risk of readmission.^18^ Failure to account for confounding by the baby's condition at birth could therefore lead to bias, i.e. the incorrect inference that shorter LOS is unrelated to increased risk of readmission. Such reverse causation has largely been ignored in studies evaluating the relationship between newborn LOS and readmission.

There are a number of ways in which this methodological challenge has been addressed. Studies from North America evaluated trends in average LOS and readmission rates over time, either through simple ecological comparisons,^10^ time series analyses evaluating the impact of strategies to reduce LOS,^17^, ^19^, ^20^ or decomposition methods assessing the proportion of neonatal admissions attributed to changes in LOS.^21^ An Australian study evaluated changes in maternal LOS and maternal readmission rates.^22^ Such aggregation over time overcomes confounding on an individual‐level, but may be subject to bias from other time‐varying exposures.^23^ Other studies have used propensity score analysis in an attempt to mimic a randomised assignment of LOS to infants matched on all other characteristics, or have tried to account for unmeasured confounding using instrumental variables.^18^, ^24^ For example, birth hour could be used as an instrument for LOS, under the assumption that birth hour is correlated with LOS but does not directly influence readmission risk.^25^

There is a lack of robust evidence for current postpartum practice on newborn LOS, particularly for safe discharge of babies born early term (37–38 weeks’ gestation) or late preterm (34–36 weeks’ gestation), who are at particularly high risk of early readmission for jaundice or feeding problems.^3^, ^4^, ^5^ We therefore explored four approaches for modelling the association between newborn LOS and risk of readmission, and assessed the impact of unmeasured confounding by clinical condition after birth. The methods included (i) modelling hospital‐level LOS and readmission rates; (ii) comparing trends over time in LOS and readmission; (iii) modelling individual LOS and risk of readmission whilst adjusting for neonatal risk factors; and (iv) instrumental variable analyses.

## Methods

Data on inpatient admissions were extracted from Hospital Episode Statistics (HES), an administrative database holding information for all admissions to National Health Service (NHS) hospitals in England.^26^ Admission records contain clinical diagnoses coded using the International Statistical Classification of Diseases and Related Health Problems 10th Revision (ICD‐10).

### Population

The study population was drawn from a linked cohort of mothers and babies whose deliveries were captured in HES and whose postnatal discharge occurred between April 2005 and February 2014. The linked cohort has been described elsewhere, and is nationally representative of key birth characteristics.^27^ Linkage success was 98% in 2012, and slightly lower in earlier years (94% in 2005).

Focussing on relatively healthy babies with a low risk of readmission (for whom small differences in length of stay might have an impact), we restricted our population to singleton births ≥34 completed weeks’ gestation, who were not admitted for neonatal intensive care, and who did not have congenital anomalies (see Table S1 for ICD‐10 diagnosis code lists). We restricted our analyses to babies with a newborn LOS ≤5 days, as most babies in England are discharged within 2 days of birth (vaginal births) or 4 days (caesarean deliveries). To allow sufficient numbers to stratify by hospital and gestational age group, we further restricted our population to hospitals with >100 births per year.

### Outcome

The primary outcome was unplanned readmission to any hospital in HES, occurring within 30 days of postnatal discharge to home. Readmissions were defined as unplanned based on the method of admission coded within HES. Transfers between hospitals were not counted as readmissions, and we considered admissions starting the day after postnatal discharge as being related to the birth admission; readmissions were defined as episodes of care starting at least 2 days following postnatal discharge. Since death is a competing risk for readmission, babies who died within 30 days of postnatal discharge were modelled as having the outcome.

### Risk factors

Newborn LOS was derived as the number of days between birth and discharge (babies discharged on the same day as birth had a 0 day LOS). Time of birth/discharge was not available in HES. Gestational age in completed weeks was based on menstrual dates or ultrasound. Babies were categorised as full term (≥39 completed weeks’ gestation), early term (37–38 weeks) or late preterm (34–36 weeks).

Small or large for gestation (<10th or >90th percentile of birthweight for gestation) was defined according to national percentiles.^28^ Delivery by caesarean, ethnic group, sex, multiple birth, maternal age, parity, year of discharge were considered as potential risk factors. Quintiles of deprivation were derived from the Index of Multiple Deprivation (IMD), based on patient postcode. On the basis of previous studies, we also derived a number of neonatal, delivery and pregnancy related conditions using diagnosis codes occurring in any field during pregnancy or the birth episode (Table S1).^29^, ^30^

### Statistical analyses

#### Methodological approaches

Based on methodological approaches used to assess the relationship between newborn LOS and readmission described in previous literature, we explored four broad methodological approaches applied to the same dataset (Table 1). The first approach, aggregated hospital‐level model, used hospital‐level mean LOS as the exposure, and aimed to avoid confounding between LOS and individual health status at birth by aggregating LOS and individual‐level risk factors to the hospital level. We hypothesised that this approach would avoid unmeasured confounding by individual health status at birth, but expected that aggregation would lead to a loss of power to detect any true association.

**Table 1:** Methodological approaches used to assess the relationship between newborn length of stay and risk of readmission

Methodological approach	Outcome	Exposure	Covariates	Number of observations^a^	Assumptions	Pros/Cons
Aggregate model						
Hospital‐level model	Hospital‐level mean risk of readmission	Hospital‐level mean newborn LOS	Risk factors (Table 2) aggregated to hospital level	Three possible gestational age groups within 142 hospitals; *n *=* *421 for vaginal / 412 for caesarean births	No unmeasured confounding between newborn LOS and risk of readmission at the hospital level	+ Exploits the fact that there will be systematic differences in hospital‐level mean LOS after adjusting for case‐mix; individual health status at birth should be unrelated to hospital‐level mean LOS − Loss of power due to aggregation
Ecological model						
Trends in LOS and risk of readmission	i) Newborn LOS; ii) Individual risk of readmission	Quarter‐year of admission (April‐June 2005 to January‐March 2014)	Individual‐level risk factors (Table 2)	*n *=* *3 791 205 babies; mean = 129 665 babies per quarter‐year	No other time‐varying factors affected LOS or admission rates during the study period	+ Natural experiment exploiting changes in discharge practices over time (as seen in other countries); aggregating over time overcomes confounding by individual health status − May be other unmeasured factors influencing trends over time (e.g. changes in breast‐feeding rates or maternal age)
Individual‐level LOS models						
Individual LOS	Individual risk of readmission	Individual newborn LOS (plus quadratic and cubic terms to assess non‐linear relationships)	Individual‐level risk factors (Table 2)	*n *=* *3 791 205 babies	No unmeasured confounding after adjusting for individual‐level risk factors	+ Power to detect association between individual LOS and risk of readmission − May be confounding between newborn LOS and risk of readmission due to unmeasured health status at birth
Deviation from expected LOS	Individual risk of readmission	Deviation between observed and expected LOS, categorised as i) shorter than expected LOS; ii) expected LOS; or iii) longer than expected LOS.	Individual‐level risk factors (Table 2)	*n *=* *3 791 205 babies	Expected newborn LOS can be predicted from individual risk factors; deviation from expected LOS reflects factors unrelated to health status	+ Exploits the fact that some babies have longer newborn LOS for reasons unrelated to their condition at birth (e.g. time of birth) − May be unable to accurately predict newborn LOS from available risk factors; may be residual confounding
Instrumental variable models						
Hospital‐level mean LOS	Individual risk of readmission	Hospital‐level mean newborn LOS (stratified by gestational age group)	Individual‐level risk factors (Table 2)	*n *=* *3 791 205 babies	Hospital‐level mean LOS is associated with individual LOS; there is no association between hospital‐level mean LOS and readmission other than through LOS; there is no additional unmeasured confounding between hospital‐level mean LOS and readmission	+ Exploits the fact that some hospitals may systematically discharge babies later; mimics experimental design to overcome confounding between individual LOS and risk of readmission − Instrumental variable may be weak (i.e. weak relationship between hospital‐level mean LOS and individual LOS); not possible to check for additional unmeasured confounding between hospital‐level mean LOS and individual health status
Number of births on the same day	Individual risk of readmission	Daily number of births within each hospital (as a proxy for bed space), categorised as fewer or greater than usual (binary variable based on ratio of number of births to mean number of births). A sensitivity analysis defined the exposure as the mean of the number of births on the day before birth, the day of birth, and the day after birth for each baby.	Individual‐level risk factors (Table 2)	*n *=* *3 791 205 babies	Daily number of births is associated with LOS; there is no association between daily number of births and readmission other than through LOS; there is no additional unmeasured confounding between daily number of births and readmission.	+ Exploits the fact that the number of births on the same day may influence discharge practices; mimics experimental design to overcome confounding between individual LOS and risk of readmission − Instrumental variable may be weak (i.e. weak relationship daily number of births and individual LOS); not possible to check for additional unmeasured confounding between daily number of births and individual health status

LOS, length of hospital stay

a The primary analysis included observations with complete data only

The second approach, ecological comparisons of time trends in LOS and risk of readmission, aimed to avoid confounding between LOS and individual health status at birth by aggregating by time and exploring trends in LOS and readmission. This approach assumes that differences in LOS practice over time are unrelated to differences in individual health status. We hypothesised that observed associations could be affected by other unmeasured factors contributing to changes in LOS or readmission rates over time.

The third approach, individual‐level LOS models, assumed that individual‐level characteristics captured in‐hospital records were sufficient to control for health status at birth and used individual LOS or deviation from expected LOS as the exposure (Table 1). We hypothesised that these models may still be confounded by health status at birth, and expected to see a positive relationship between LOS and risk of readmission (babies with longer LOS are sicker and more likely to be readmitted).

The fourth approach, instrumental variable models, attempts to use an alternative ‘latent’ variable as a proxy for individual LOS, which is otherwise unrelated to risk of readmission. The instrumental variables were daily number of births and hospital‐level mean LOS. We hypothesised that this approach would be the least prone to unmeasured confounding.

Since confounding by the baby's condition at birth would bias results towards longer LOS being associated with greater risk of readmission, we assumed that where a negative relationship was observed, this was likely to reflect a true association between increased LOS and reduced risk of readmission. Assumptions and limitations of the different approaches are detailed in Table 1.

#### Models

For all models predicting risk of readmission, we used Poisson generalised linear models with a log link. To predict expected LOS (and LOS trends), we compared Poisson, negative Binomial and linear regression models. Inspection of model residuals and deviance statistics indicated that Poisson generalised linear models provided the best fit.^31^ All models used robust standard errors to allow for clustering of observations within hospitals and included interaction terms for LOS and gestational age group (full term, early term, late preterm).

For the model defining the exposure as deviation from expected LOS (approach 3), we created three categories (shorter than expected, expected, and longer than expected), to aid interpretation of results. Similarly, for the instrumental variable approach incorporating the daily number of births, we categorised the exposure as a binary variable (greater or fewer births than usual). As a sensitivity analysis, for both these models, we defined the exposure as a continuous variable rather than categorical.

For instrumental variables to be valid for LOS, the instrument should be associated with LOS; there should be no association between the instrument and readmission other than through LOS; there should be no additional unmeasured confounding between the instrument and readmission. The first two of these assumptions were tested using linear and logistic regression, respectively; we did not test the third assumption as there was no reason to suspect unmeasured confounding between the instruments and readmission.

The primary analysis was based on a complete case analysis. However, to account for missing values in gestation or birthweight, or where there were suspected coding errors (birthweight >4 standard deviations from the median according to published reference values), we conducted a sensitivity analysis using multiple imputation by chained equations (further details and results presented in Appendix S1).^28^

## Results

**Figure 1:**
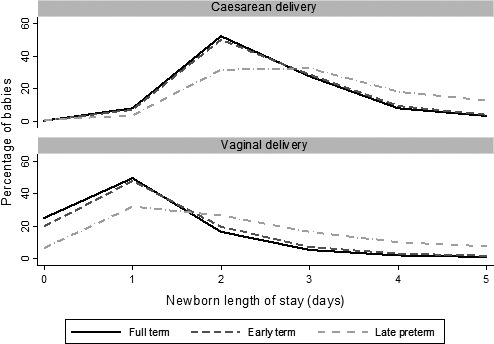
Distribution of newborn length of stay for babies in the study population, by gestational age (full term, 39 +  completed weeks’; early term, 37–38 completed weeks’; late preterm, 34–36 completed weeks’).

**Figure 2:**
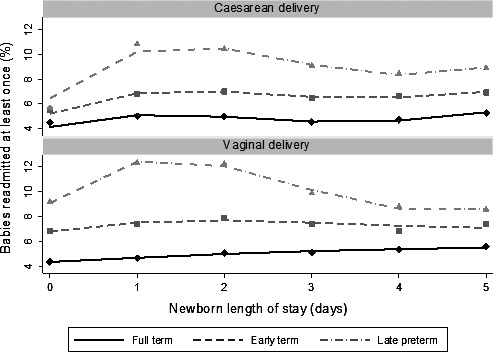
Relationship between the percentage of babies with one or more unplanned readmissions and newborn LOS, by gestational age (full term, 39 +  completed weeks’; early term, 37–38 completed weeks’; late preterm, 34–36 completed weeks’). Symbols represent observed values, and line represents model values. The percentage of caesarean births with a newborn LOS of 0 days was very small (0.4%), but this category has been included for completeness.

The median newborn length of stay was 1 day (2 days for late preterm babies, Figure 1). The majority (90%) of births were discharged within 2 days of vaginal birth or 4 days of birth by caesarean section. Overall, 5.2% (*n *=* *244 827) of babies in the study population had one or more unplanned readmissions within 30 days post‐discharge (7.2% for early term, 10.6% for late preterm births). Characteristics are shown in Table 2. Risk of readmission tended to increase with longer newborn LOS (Figure 2), suggesting that, before adjusting for any risk factors, newborn LOS reflects the underlying health condition at birth.

**Table 2:** Study population characteristics^a^

	No readmission (*N *=* *3 988 745)		Readmission (*N *=* *216 780)		Readmission Adjusted OR (95% CI)
	*n*	%	*n*	%	
Birth by caesarean	805 319	20.2	46 518	21.5	1.00 (0.98, 1.02)
Gestational age at birth					
Full term	2 917 618	80.2	144 619	71.1	1.00 (Reference)
Early term	642 788	17.7	49 900	24.5	1.57 (1.54, 1.59)
Late preterm	76 068	2.1	8993	4.4	2.37 (2.27, 2.48)
Missing	786 346	19.7	41 618	19.2	–
Size for gestation					
Small (<10th percentile)	279 654	7.8	15 525	7.7	1.01 (1.00, 1.03)
Normal	2 933 635	81.7	162 672	80.9	1.00 (Reference)
Large (>90th percentile)	376 808	10.5	22 911	11.4	1.09 (1.07, 1.11)
Missing	832 723	20.9	44 022	20.3	–
Female sex					
Female	2 192 547	55.0	111 301	51.3	0.85 (0.84, 0.86)
Deprivation quintile					
Most deprived	1 202 065	27.4	73 176	30.1	0.92 (0.88, 0.96)
2	968 462	22.1	53 461	22.0	0.90 (0.85, 0.95)
3	807 094	18.4	43 353	17.8	0.90 (0.84, 0.95)
4	711 692	16.2	37 640	15.5	0.88 (0.81, 0.95)
Least deprived	692 885	15.8	35 251	14.5	1.00 (Reference)
Missing	33 102	0.8	2249	1.0	–
Ethnic group					
White	3 248 134	81.4	182 344	84.1	1.00 (Reference)
Mixed	167 072	4.2	9391	4.3	0.96 (0.91, 1.01)
Asian	458 088	11.5	29 350	13.5	1.08 (1.01, 1.17)
Black	237 402	6.0	9953	4.6	0.72 (0.66, 0.79)
Other	146 633	3.7	8164	3.8	0.98 (0.90, 1.07)
Unknown	165 491	4.1	5928	2.7	0.69 (0.61, 0.78)
Maternal age (years)					
≤18	143 989	3.3	9586	3.9	0.93 (0.90, 0.95)
19–24	943 022	21.4	57 259	23.4	0.86 (0.83, 0.89)
25–29	1 210 918	27.4	67 669	27.6	1.00 (Reference)
30–34	1 260 231	28.5	65 848	26.9	0.82 (0.79, 0.85)
35–39	699 717	15.8	35 729	14.6	0.81 (0.77, 0.85)
≥40	157 776	3.6	8652	3.5	0.84 (0.79, 0.90)
Missing	7167	0.2	387	0.2	–
Primiparous mother	1 833 003	46.0	106 972	49.3	1.1 (1.07, 1.14)
Perinatal infection^b^	26 824	0.7	1903	0.9	1.18 (1.11, 1.26)
Pregnancy risk factor^b^	386 428	9.7	24 912	11.5	1.05 (1.03, 1.07)
Delivery risk factor^b^	357 418	9.0	21 325	9.8	1.07 (1.02, 1.13)
Neonatal risk factor^b^	2209	0.1	220	0.1	1.75 (1.23, 2.50)
Conditions related to preterm birth (<37 weeks) b	1807	0.0	219	0.1	0.96 (0.83, 1.10)
Substance‐related risk factor^b^	3763	0.1	305	0.1	1.21 (0.79, 1.84)
Season of birth					
January–March	1 036 502	26.0	57 352	26.5	1.00 (Reference)
April–June	1 103 980	27.7	58 826	27.1	0.96 (0.94, 0.97)
July–September	1 163 600	29.2	61 433	28.3	0.94 (0.92, 0.96)
October–December	1 118 738	28.0	67 519	31.1	1.09 (1.07, 1.10)

a Exclusions were multiple births, babies admitted for neonatal intensive care, congenital anomalies, <34 weeks’ gestation, and newborn LOS >5 days.

b Descriptions and code lists provided in Table S1.

### Aggregate model

There was no association between hospital‐level mean LOS and risk of readmission for vaginal births (RR 0.87, 95% CI 0.66, 1.13) and caesarean births (RR 0.89, 95% CI 0.72, 1.12).

### Ecological model

Between April 2005 and February 2014, newborn LOS for vaginal births decreased by 2.0% annually: median LOS fell from 1.4 days in 2005 to 1.2 days in 2014 and this was consistent across gestational age groups. Over the same period, risk of readmission increased by 4.4% annually (from 4.4 in 2005 to 6.3% in 2014) and the increase was greater in early term (5.6%) and late preterm births (4.5%).

For caesarean births, newborn LOS decreased by 3.4% annually: median LOS fell from 2.9 days in 2005 to 2.2 days in 2014 and this was consistent across gestational age groups. The risk of readmission increased by 5.1% annually (from 4.6 in 2005 to 6.3 in 2014), and the increase was greater in early term (5.3%) and late preterm births (5.9%).

### Individual‐level LOS models

For vaginal births, each additional day of newborn stay was associated with a 3.0% (95% CI 1.9, 4.2) increase in the adjusted risk of readmission. However, the association was reversed for late preterm babies, for whom each additional day of newborn stay was associated with an 8.6% (95% CI 6.1, 10.5) decreased risk of readmission (Figure 2). For caesarean births, there was no linear association between individual LOS and readmission (aRR 1.01, 95% CI 0.99, 1.04). However, inclusion of quadratic and cubic terms suggested a non‐linear relationship for both groups (Figure 2).

Positive associations between newborn LOS and risk of readmission suggest that confounding by individual health status after birth remains, even after adjusting for individual risk factors. However, since this confounding would bias results towards no association, the association between longer LOS and decreased risk of readmission for late preterm births is likely to be true.

### Deviation from expected LOS

**Figure 3:**
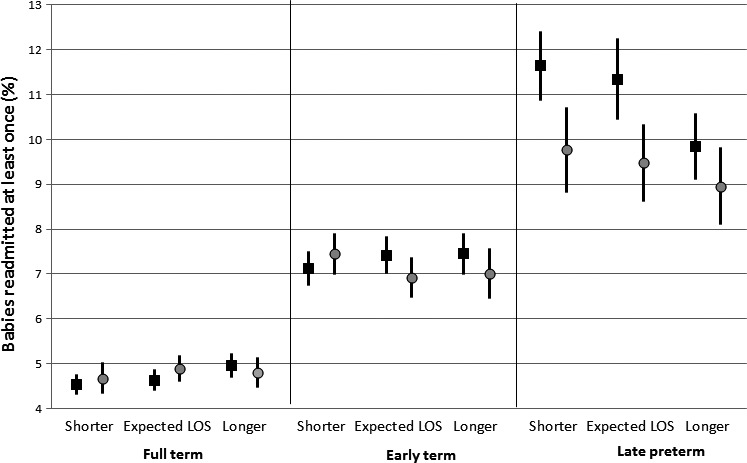
Risk of readmission and ratio of observed/expected LOS by method of delivery (vaginal = squares; caesarean = circles) and gestational age (full term, 39 +  completed weeks’; early term, 37–38 completed weeks’; late preterm, 34–36 completed weeks’). Expected LOS ratio = 0.77–1.13; shorter than expected LOS ratio = 0.00–0.77; longer than expected LOS ratio = 1.13–7.26.

Vaginal births with shorter than expected LOS had a 2.4% (95% CI 0.5, 4.4) decreased risk of readmission, and those with longer than expected LOS had a 4.9% (95% CI 2.9, 6.9) increased risk of readmission, compared with babies in the expected LOS category. Patterns were again reversed for late preterm babies: those with longer LOS than expected had a 14.9% (95% CI 7.7, 21.6) decreased risk of readmission (Figure 3). Similar patterns were seen for caesarean births, although effect sizes were smaller (Figure 3). The sensitivity analysis treating deviation from expected LOS as a continuous variable showed similar results.

### Instrumental variable models

#### Hospital‐level mean LOS

Overall, the hospital‐level mean LOS was 1.5 days (median 1.4 days, interquartile range 1.3–1.6), ranging from 1.4 days for full term babies to 2.4 days for late preterm babies. There was some variation by hospital (Figure S1). Two tests indicated that hospital‐level mean LOS was a valid instrument for individual LOS: hospital‐level mean LOS was associated with individual LOS (mean LOS increased by 1 day for each day increase in hospital‐level mean LOS); hospital‐level mean LOS was not associated with risk of readmission after adjusting for individual LOS (RR 0.96, 95% CI 0.84, 1.14).

There was no association between hospital‐average LOS and risk of readmission for both vaginal and caesarean births. However, an association was observed for late preterm, vaginal births. For this group, hospitals with longer mean LOS were associated with a lower risk of readmission: each additional day of hospital‐level mean LOS decreased the risk of readmission for late preterm babies by 11.7% (95% CI 1.3, 20.0). This corresponds to a 1.4% absolute difference in the percentage of late preterm babies readmitted comparing hospitals with a mean LOS of 2 vs. 3 days (11.4 vs. 10.0% babies readmitted).

#### Number of births on the same day

Two tests indicated that the number of births on the same day was a valid instrument for individual LOS: the instrumental variable was weakly associated with LOS (LOS was 0.01 days shorter (95% CI 0.01, 0.02) when there were a greater number of births than usual); the number of births was not associated with readmission after adjusting for newborn LOS (RR 1.01, 95% CI 0.99, 1.02).

There was no evidence for an association between a greater number of births and overall risk of readmission for either vaginal births or caesarean births and no differences were seen by gestational age. No differences were seen in the sensitivity analyses using the mean number of births over 3 days as the instrumental variable, or when using the number of births as a continuous variable. These findings suggest that on days with a greater than usual number of births, LOS tends to be shorter, but this did not result in increased readmissions. The lack of observed association between number of births and LOS could be due to the weak strength of the instrumental variable.

## Comment

We evaluated the relationship between newborn LOS and risk of readmission within 30 days post‐discharge using population‐level data from over 4 million births in English hospitals. We used a number of different methodological approaches to examine this issue, because it was unclear whether variables captured in individual‐level administrative data sufficiently accounted for confounding by the baby's condition at birth. The results indicate that the relationship is dependent upon the methodological approach used. First, analysis of hospital‐aggregated readmission rates and mean LOS, although reducing statistical power, suggested that longer newborn LOS was associated with a decreased risk of readmission. Similarly, analysis of trends over time suggested that decreasing LOS coincided with increased readmission rates. However, trends in readmission rates differed for late preterm babies, whereas trends in LOS were consistent across gestational age groups, suggesting that decreasing LOS over time is not the only factor contributing to rising readmission rates. Finally, modelling individual LOS provided no evidence of an association between LOS and risk of readmission overall, but consistently showed a decreased risk of readmission for late preterm babies with longer newborn LOS, particularly for vaginal births.

Results from previous studies based on modelling individual newborn LOS are conflicting, and do not address differences by gestational age.^3^ Our finding of differential associations between newborn LOS and risk of readmission according to gestational age has two possible explanations. First, early discharge may be safe for full term and early term babies, but not for late preterm babies. Alternatively, benefits of longer LOS may exist for full term and early term babies (as well as for late preterm babies), but the association remains obscured due to unmeasured confounding by the baby's condition at birth. Although the methodological approaches were able to overcome some of this confounding, any remaining confounding would bias effects to the null. This means that the benefits of longer newborn LOS and lower risk of readmission observed for late preterm babies in this study are likely to be under‐estimated, and that weaker relationships for more mature babies could still be obscured. In both cases, the finding for late preterm babies is likely due to a stronger effect for this more vulnerable group.

The association between late preterm birth and early readmission (particularly for jaundice and feeding problems) is well recognised, and has been related to an ‘unreadiness’ at the time of discharge for apparently healthy but immature babies.^32^, ^33^ In particular, babies who are discharged before symptoms appear (typically at 2 or 3 days after birth for jaundice) are often readmitted.^34^ Although recommendations for safe discharge of late preterm infants exist internationally, there are no national guidelines for postnatal LOS for late preterm babies in the United Kingdom, and local practices vary.^35^, ^36^ This study was conducted in a period after publication of guidance from the American Academy of Pediatrics, suggesting that recognised best practice for this group has not been effective, or effectively utilised, in the UK context.^37^

### Strengths and limitations of the study

A major strength of the study is the large sample size and the use of a population‐level data source containing information on both maternal and neonatal risk factors. However, our results highlight that adequate control for severity of illness through case‐mix adjustment using only data captured in hospital administrative data can be difficult, even when considering detailed information coded in diagnosis fields for both mothers and babies. Results from modelling individual LOS exposure demonstrated patterns of increasing risk of readmission by increasing LOS, suggesting that unmeasured confounding by severity of condition after birth remained, even after controlling for a number of neonatal and maternal risk factors. This study was limited by a lack of more detailed information on potential confounders for severity of condition after birth (e.g. Apgar score), time of birth and discharge, complete recording of admission to neonatal intensive care or special care baby units, and other postnatal confounders such as breast feeding.^34^ However, these results were robust in sensitivity analyses using multiple imputation for missing gestation or birthweight (Appendix S1). Although we adjusted for deprivation, we were unable to take into account social risk factors for readmission such as smoking. We were also unable to capture the small proportion of births (<3%) or readmissions that occurred outside of the NHS setting.^38^

Future research could use sub‐national data to help understand the causes of variation in LOS between hospitals, based on information on availability of local services within and outside the hospital (e.g. use of emergency departments, paediatric admission units, outreach neonatal nurses and timing of midwifery and health visitor support).^39^, ^40^ Further subgroup analysis could be used to identify subgroups with different effects. For example, first time teenage mothers may benefit more than older mothers from increased newborn LOS.^41^

## Conclusions

Cautious interpretation of our results indicates that discharge policies for term babies may not be appropriate for those born a few weeks too early, and that increased in‐hospital support may benefit late preterm babies who are at increased risk of potentially preventable readmissions.^36^, ^37^ Ultimately, the balance between intensity of in‐hospital maternity care and frequency, timing and duration of follow‐up visits should be based on individual and local needs.^42^ Researchers using observational data to evaluate relationships between LOS and risk of readmission – irrespective of specialty – should be aware of the risk of confounding when modelling individual‐level exposure, and should explore different methodological approaches to account for this confounding.

## Supporting information


**Figure S1.** Variation in newborn LOS by hospital and gestational age (full term, 39 +  weeks; early term, 37–38 weeks; late preterm, 34–36 weeks).

**Table S1.** ICD 10 code lists for risk‐factor groups and exclusion criteria.

**Appendix S1.** Results of sensitivity analysis using multiple imputation for missing values of birthweight, gestation, Index of Multiple Deprivation or maternal age.
