# Supplementary figures and images for: Newborn Length of Stay and Risk of Readmission
Source: Paediatr Perinat Epidemiol. 2017 Apr 18;31(3):221–32. doi: 10.1111/ppe.12359 (PMC5518288; doi:10.1111/ppe.12359)

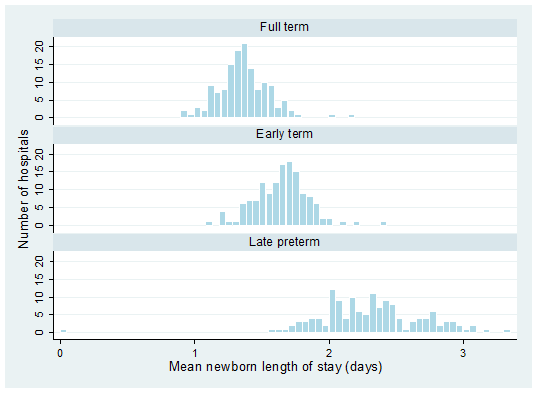

Supplement: Supplementary file 1 — Figure S1. Variation in newborn LOS by hospital and gestational age (full term, 39 + weeks; early term, 37–38 weeks; late preterm, 34–36 weeks). [file PPE-31-221-s001.tif]
