# Supplementary material for: Newborn Length of Stay and Risk of Readmission
Source: Paediatr Perinat Epidemiol. 2017 Apr 18;31(3):221–32. doi: 10.1111/ppe.12359 (PMC5518288; doi:10.1111/ppe.12359)
Supplement: Supplementary file 2 — Table S1. ICD 10 code lists for risk‐factor groups and exclusion criteria. [file PPE-31-221-s002.docx]

## **eTable 1: ICD 10 code lists for risk-factor groups and exclusion criteria**

| **Risk factor group** | **Description** | **ICD10 codes** |
| --- | --- | --- |
| Conditions related to preterm birth* | Necrotising enterocolitis | P77 |
|  | Intraventricular hemorrhage | P52, P912 |
|  | Retinopathy of prematurity | H351 |
|  | Respiratory distress syndrome | P22 |
| Delivery risk factors | Birth trauma | P10-P15 |
|  | Complications of delivery | P03 |
|  | Hypoxia | P20-P21 |
|  | Amniotic fluid embolism | O881 |
|  | Chorioamnionitis | O411 P027-P029 |
|  | Umbilical cord problem | P020, P024-P026 |
|  | Fetal hemorrhage | P50, P51, P53, P54 |
|  | Maternal hemorrhage | O430 |
|  | Umbilical cord prolapse | O69 |
| Neonatal medical conditions | Complex chronic conditions | B20-B23, D55, D561, D562, D570-D572, D58, D80-D84, D898, D899, E343, E70-E730, E74, E76-E79, E803-E807, E83-E85, E881, E882, E888, E889, F70, F72, F73, F842, G10-G12, G20, G23, G240-G242, G248, G250-G256, G318, G319, G40, G41, G71, G72, G80-G82, G901, G903, G904, G91, G940-G942, G95, G99, I42, I44, I45, I47-I49, I515, K44, K50-K51, K73-K74, K754, K758-K760, M41, N18, P27, P90 |
| Substance-related risk factors | Neonatal abstinence syndrome | P961 |
|  | Noxious influences | P04 |
| Perinatal infection | Perinatal infection | P35-P39 |
|  | Meningitis or encephalitis | G00-G09 |
| Pregnancy risk-factors | Intrauterine fetal death | O364, P95 |
|  | Eclampsia | O14,O15 |
|  | Gestational hypertension | O13,O16 |
|  | Placental abruption or infarction | O45,O431, O438, O439 |
|  | Uterine rupture | O710, O711 |
|  | Diabetes in pregnancy** | P700, O24, E10-E14 |
| Congenital anomaly |  | Q00-Q07, Q10.4, Q10.7, Q11-Q12, Q13.0-Q13.4, Q13.8, Q13.9, Q14-Q16, Q20-Q26, Q18.8, Q30-Q37, Q38.0, Q38.3, Q38.4, Q38.6-Q38.8, Q39, Q40.2, Q40.3, Q40.8, Q40.9, Q41, Q42, Q43.1, Q43.3-Q43.7, Q43.9, Q44, Q45, Q50.0, Q51, Q52.0-Q52.2, Q52.4, Q54.0-Q54.3, Q54.8, Q54.9, Q55.0, Q55.5, Q56, Q60.1, Q60.2, Q60.4-Q60.6, Q61, Q62.0-Q62.6, Q62.8, Q63.0-Q63.2, Q63.8, Q63.9, Q64, Q65.0-Q65.2, Q65.8, Q65.9, Q67.5, Q68.2, Q68.3-Q68.5, Q71-Q73, Q74, Q75.0, Q75.1, Q75.3-Q75.9, Q76.1-Q76.4, Q77, Q78, Q79.0, Q79.2-Q79.5, Q79.6, Q79.8, Q82.0-Q82.4, Q82.9, Q86.2, Q85, Q86.0, Q86.1, Q86.8, Q87.8, Q89.1, Q89.2, Q89.3, Q89.7-Q89.9, Q90-Q93, Q95.2, Q95.3, Q97, Q99 |

* restricted to babies born <37 weeks’ completed gestation; ** recorded during pregnancy
