# Supplementary material for: Newborn Length of Stay and Risk of Readmission
Source: Paediatr Perinat Epidemiol. 2017 Apr 18;31(3):221–32. doi: 10.1111/ppe.12359 (PMC5518288; doi:10.1111/ppe.12359)
Supplement: Supplementary file 3 — Appendix S1. Results of sensitivity analysis using multiple imputation for missing values of birthweight, gestation, Index of Multiple Deprivation or maternal age. [file PPE-31-221-s003.docx]

**eAppendix: Sensitivity analysis using multiple imputation for missing values of gestation, size for gestation, IMD or maternal age**

To account for missing data on gestational age (n=827,964; 19.7%), size for gestation (n=876,964; 20.8%), maternal age (n=7554; 0.2%) and Index of Multiple Deprivation (n=35,351; 0.8%), multiple imputation using chained equations without replacement was performed using Stata 14 (Release 14. StataCorp LP). Multiple imputation was used for the ecological model approach, the individual LOS approach, and the instrumental variables approach, but not the aggregated model.

The imputation model included mode of delivery, ethnic group, parity, newborn length of stay, along with risk factors relating to delivery, prematurity, neonatal medical conditions, substance use, perinatal infection, and pregnancy (see eTable 1 for details). We also included the outcome (readmission or death) in the imputation model. Results were combined from five imputed datasets.

Results were almost identical to the complete case analysis.

*Ecological model*

For vaginal births, newborn LOS decreased by 2.2% per year between April 2005 and February 2014. The risk of readmission increased by 4.4% per year, and the increase was greater in early term (5.7%) and late preterm births (4.6%).

For caesarean births, newborn LOS decreased by 3.4% per year. The risk of readmission increased by 5.1% per year, and the increase was greater in early term (5.3%) and late preterm births (5.6%).

### *Individual-level LOS models*

##### Individual LOS

For vaginal births, each additional day of newborn stay was associated with a 3.3% (95% CI 2.1%, 4.5%) increase in the adjusted risk of readmission. However, the association was reversed for late preterm babies, for whom each additional day of newborn stay was associated with an 8.5% (95% CI 6.2%, 10.8%) *decreased* risk of readmission. For caesarean births, there was no linear association between individual LOS and readmission (aRR 1.01; 95% CI 0.99, 1.04).

##### Deviation from expected LOS

Vaginal births with shorter than expected LOS had a 2.0% (95% CI 0.4%, 4.1%) decreased risk of readmission, and those with longer than expected LOS had a 4.6% (95% CI 2.8%, 6.5%) increased risk of readmission, compared with babies in the expected LOS category. For late preterm babies, those with longer LOS than expected had a 13.2% (95% CI 6.7%, 19.4%) *decreased* risk of readmission. Similar patterns were seen for caesarean births.

### *Instrumental variable models*

##### Hospital-level mean LOS

There was no association between hospital-average LOS and risk of readmission (aRR 1.01; 95% CI 0.88, 1.16) for vaginal births; 0.81 (95% CI 0.66, 1.01) for caesareans). For late preterm, vaginal births, hospitals with longer mean LOS were associated with a lower risk of readmission: each additional day in hospital-level mean LOS decreased the risk of readmission by 12.0% (95% CI 1.7, 21.3%)).

##### Number of births on the same day

There was no evidence for an association between a greater number of births and overall risk of readmission for either vaginal births or caesareans.
